# Two-layer analytical model for estimation of layer thickness and flow using Diffuse Correlation Spectroscopy

**DOI:** 10.1371/journal.pone.0274258

**Published:** 2022-09-16

**Authors:** Jingyi Wu, Syeda Tabassum, William L. Brown, Sossena Wood, Jason Yang, Jana M. Kainerstorfer

**Affiliations:** 1 Department of Biomedical Engineering, Carnegie Mellon University, Pittsburgh, Pennsylvania, United States of America; 2 Neuroscience Institute, Carnegie Mellon University, Pittsburgh, Pennsylvania, United States of America; Politecnico di Milano, ITALY

## Abstract

Diffuse correlation spectroscopy (DCS) has been widely explored for its ability to measure cerebral blood flow (CBF), however, mostly under the assumption that the human head is homogenous. In addition to CBF, knowledge of extracerebral layers, such as skull thickness, can be informative and crucial for patient with brain complications such as traumatic brain injuries. To bridge the gap, this study explored the feasibility of simultaneously extracting skull thickness and flow in the cortex layer using DCS. We validated a two-layer analytical model that assumed the skull as top layer with a finite thickness and the brain cortex as bottom layer with semi-infinite geometry. The model fitted for thickness of the top layer and flow of the bottom layer, while assumed other parameters as constant. The accuracy of the two-layer model was tested against the conventional single-layer model using measurements from custom made two-layer phantoms mimicking skull and brain. We found that the fitted top layer thickness at each source detector (SD) distance is correlated with the expected thickness. For the fitted bottom layer flow, the two-layer model fits relatively consistent flow across all top layer thicknesses. In comparison, the conventional one-layer model increasingly underestimates the bottom layer flow as top layer thickness increases. The overall accuracy of estimating first layer thickness and flow depends on the SD distance in relationship to first layer thickness. Lastly, we quantified the influence of uncertainties in the optical properties of each layer. We found that uncertainties in the optical properties only mildly influence the fitted thickness and flow. In this work we demonstrate the feasibility of simultaneously extracting of layer thickness and flow using a two-layer DCS model. Findings from this work may introduce a robust and cost-effective approach towards simultaneous bedside assessment of skull thickness and cerebral blood flow.

## Introduction

Microvascular blood flow ensures delivery of oxygen and nutrients to the tissue and subsequent removal of metabolic by-products from the tissue, hence crucial for healthy functionality of tissue and organs. On the other hand, abnormal blood flow in the vasculature were shown to be associated with critical pathologies such as cardiovascular diseases, stroke, head trauma, peripheral arterial disease, cancer, and other conditions where the impaired blood flow impacts surrounding tissue [1–3]. As a result, continuous monitoring of blood flow is crucial, and depending on the clinical need, physicians currently use a variety of non-invasive techniques to monitor microvascular blood flow such as Magnetic Resonance Imaging (MRI), Positron Emission Tomography (PET), Computed Tomography (CT), etc [4–7]. However, the currently available tools aren’t suitable for continuous blood flow monitoring as they entail major disadvantages including discomfort to the patient, harm from the use of ionizing radiation and contrast agents, poor portability and high cost [8–10]. In essence, what is needed in healthcare is a non-invasive alternative which is safe, low cost, and easy to operate for long periods of time at the patient bedside.

Diffuse correlation spectroscopy (DCS) is a diffuse optical technique that utilizes harmless near-infrared light to measure deep tissue microvascular flow with the penetration depth in centimeters [2, 11–16]. Like other diffuse optical methods such as Near Infrared Spectroscopy (NIRS) and Diffuse Optical Spectroscopy (DOS), DCS is non-invasive and can be made to have a high temporal resolution [2, 17]. DCS is relatively low-cost, easily portable, and is more suitable for continuous bedside monitoring compared to other more well-known non-invasive vascular imaging techniques such as MRI and US [4–6, 8–10]. Moreover, DCS is more sensitive to the blood flow in capillaries and small vessels, making it potentially a better indicator of tissue perfusion than methods which measure blood flow velocity in large vessels such as US [18].

Conventionally, in continuous-wave DCS (CW-DCS), a long-coherence-length laser is used to acquire local blood flow information by fitting the normalized electric field temporal autocorrelation function (*g*_1_) to the measured temporal intensity fluctuations [2]. In the simplest case, it is assumed that the tissue being measured is a homogeneous semi-infinite medium. However, most biological tissues are layered with each layer encompassing different physiological and optical properties–for DCS to measure the human brain, light must propagate through multiple layers including scalp and skull [19].

Recent studies with DCS have accounted for the layered structure in head by exploring various multi-layer analytical models. For example, Gagnon et al. proposed a two-layer analytical model which was derived from a time-domain diffusion equation developed by Kienle et al. specific to two-layered geometry [20, 21]. Using Monte Carlo (MC) simulations of the human brain and experimental validation in a two-layer phantom, Gagnon and his colleagues demonstrated that the two-layer model could distinguish between changes in the superficial layers and the brain cortex. Alternatively, for the CW domain, Li et al. proposed a three-layer analytical model and demonstrated accurate separation of cortical hemodynamics from variations in extracerebral layers such as scalp and skull in response to motor stimulations in human [22]. Few other studies with DCS have revealed the feasibility and reliability of the multi-layer analytical model [19, 23, 24]. Recent studies using MC simulations also showed that using a three-layer model can improved the accuracy of the CBF extraction [25–27].

In a recent study that implemented a MC based multi-layer model using multi-distance DCS measurements to extract changes in CBF, Carp et al. concluded that to recover accurate cortical blood flow, a precise knowledge of scalp/skull thickness is essential [28]. In addition, skull thickness is essential in determining skull deformation especially in the case of traumatic brain injury (TBI) [29]. As a result, one can assume that the information of CBF added with the knowledge of skull thickness may facilitate diagnosis and treatment strategies in care of TBI and other brain complications. In this regard, current state of the art methods for quantifying skull intactness are typically imaging modalities such as CT or US which offer many disadvantages including poor safety profile as mentioned above [8–10, 29]. In summary, continuous bedside monitoring to quantify both skull thickness and CBF is presently not available for clinical use.

Towards this goal, we adapted a two-layer analytical model from the multi-layer model described by Li et al. [22]. In this model, we assumed the skull as top layer with finite thickness and the brain cortex as bottom layer with semi-infinite geometry; we then use it to fit for thickness in the top layer thickness and flow in the bottom layer. The flow extracted from the proposed model was tested against the conventional single-layer model using measurements from custom made two-layer phantoms mimicking skull and the brain for various source-detector (SD) separations. Lastly, since layer optical properties (absorption coefficient (*μ*_*a*_) and reduced scattering coefficient ($\mu_{s}^{\prime }$)) of the phantoms were assumed in the model, an error analysis was conducted to explore how much an incorrect assumption of layer optical properties might alter the model performance. This work explores the feasibility of simultaneous extraction of layer thickness and flow, which may introduce a robust and cost-effective approach towards simultaneous bedside assessment of skull thickness and CBF.

## Methods

In this section, the theory of the single-layer and two-layer analytical models for CW-DCS are explained. In addition, details on device instrumentation, data acquisition and processing strategies are described. Lastly, details on fabrication of the two-layer phantoms and measurement are outlined.

### Single-layer analytical model

In DCS, the near-infrared (NIR) light is directed into a diffusive medium. Photons then undergo dynamic phase shifts from the moving scatterers, i.e., red blood cells (RBC) [2]. Such phase shifts give rise to temporally varying speckle patterns which contains dynamic information of the scatterers. DCS measures the temporal fluctuation of the reflected light, from which a metric for CBF can be computed. The temporal electric field autocorrelation function (*G*_1_) of the scattered electric field (***E****(t)*), which carries the dynamic properties of the scatterer, can be written as [30]:

$$G_{1}(\tau )=\langle E(t)\cdot E^{*}(t+\tau )\rangle$$ (1)

where the brackets 〈〉 denote the average over time *t*, and *τ* is the delay time.

In semi-infinite homogeneous media, *G*_1_ can be described by the correlation diffusion equation:

$$G_{1}(\rho ,\tau )=\frac{3\mu_{s}^{\prime }}{4\pi }(\frac{\exp\left(-K(\tau )r_{1}\right)}{r_{1}}-\frac{\exp\left(-K(\tau )r_{2}\right)}{r_{2}})$$ (2)

where

$$K(\tau )=\sqrt{3\mu_{a}\mu_{s}^{\prime }+\mu_{s}^{\prime 2}k_{0}^{2}\langle \Delta r^{2}(\tau )\rangle }$$ (3)

and

$$\begin{array}{c} r_{1}=\sqrt{\rho^{2}+{z_{0}}^{2}}\\ r_{2}=\sqrt{\rho^{2}+{\left(z_{0}+2z_{b}\right)}^{2}} \end{array}$$ (4)

*ρ* is the source-detector distance, $z_{0}=1/\mu_{s}^{\prime },\;z_{b}=\frac{2}{3\mu_{s}^{\prime }}\frac{1+R_{eff}}{1-R_{eff}},\;R_{eff}=-1.44n^{-2}+0.71n^{-1}+0.064n+0.668$, *n* is the refractive index, *α* describes the fraction of photon scattering events from moving scatterers such as red blood cells in the tissue, *k*_0_ is the wave-number of the light in tissue, and 〈Δ*r*^2^(*τ*)〉 is the mean square displacement of the moving scatterers. In most DCS experiments in living tissues, the 〈Δ*r*^2^(*τ*)〉 has been found to be reasonably well approximated as an effective Brownian motion, i.e., 〈Δ*r*^2^(*τ*)〉 = 6*D*_*B*_*τ*, where *D*_*B*_ is the Brownian diffusion coefficient of the moving scatterers and thus the flow dependent parameter in the correlation-diffusion equation [30].

In practice, DCS measures the fluctuations of the scattered light intensity. The normalized temporal intensity autocorrelation function is calculated as $g_{2}(\tau )=\langle I(t)I(t+\tau )\rangle /\langle I(t)\rangle^{2}$, where *I* is the measured intensity. The normalized temporal electric field autocorrelation function *g*_1_ is related to *g*_2_ by the Siegert relationship [2, 11].

$$g_{2}(\tau )=1+\beta {\left|g_{1}(\tau )\right|}^{2}$$ (5)

where $g_{1}(\tau )=\langle E(t)\cdot E^{*}(t+\tau )\rangle /\langle E(t)\cdot E^{*}(t)\rangle$, and *β* is the autocorrelation correction factor which depends on the experimental setup.

Another single-layer analytical model described by Li et al. will be introduced in the next session.

### Multi-layer analytical model

**Fig 1:**
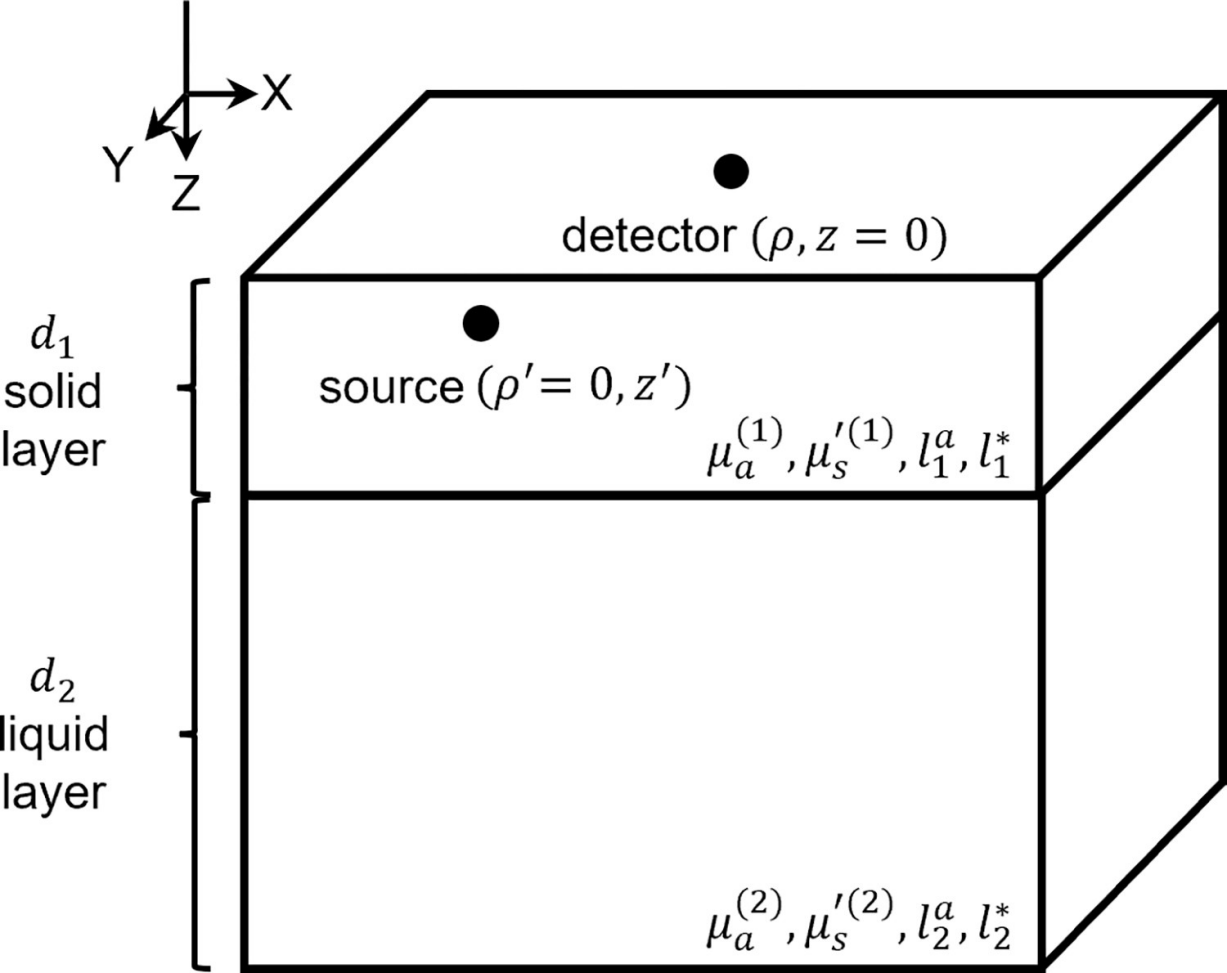
Schematic of the two-layer scattering medium including locations of the source and the detector. Here, $\mu_{a}^{\left(n\right)}$ and ${\mu^{\prime }}_{s}^{\left(n\right)}$ are the absorption and reduced scattering coefficients, $l_{n}^{*}$ is the transport mean free path, and $l_{n}^{a}$ is the absorption mean free path, for the respective layers where n = 1–2. The top layer is the solid layer with finite thickness (*d*_1_), the bottom layer is the liquid layer with semi-infinite geometry (*d*_2_).

Li et al. proposed a temporal field autocorrelation function with respect to a multi-layered tissue geometry [22]. In this manuscript, we adapted the model described by Li et al. and formulated it for a two-layer geometry to simulate the skull and the brain, as shown in Fig 1. The optical properties for each layer are represented by the transport mean free path $l_{n}^{*}=1/{\mu_{s}^{\prime }}^{\left(n\right)}$ [31] and absorption mean free path $l_{n}^{\left(a\right)}=1/\mu_{a}^{\left(n\right)}$, where $\mu_{a}^{\left(n\right)}$ and ${\mu_{s}^{\prime }}^{\left(n\right)}$ are absorption and reduced scattering coefficients for each layer with n = 1–2. The *d*_*n*_ represents thicknesses for respective layers.

The temporal field autocorrelation function $G(r,\tau )=\langle E(t)\cdot E^{*}(t+\tau )\rangle$ can be described by the correlation diffusion equation [12] as

$$\left[\nabla^{2}-\alpha_{n}^{2}(\tau )]G(r,\tau )=-s_{0}\delta (r-r^{\prime }\right)$$ (6)

where s_0_ is a point-like monochromatic light source located at ***r***′ = {***ρ***′ = 0, *z*′} inside the first layer; ***ρ*** represents the transverse coordinate, and $z^{\prime }=1/{\mu_{s}^{\prime }}^{\left(1\right)}$. The decorrelation due to the moving scatterers, *α*_*n*_, can be written as

$$\alpha_{n}(\tau )=\sqrt{\frac{3}{l_{n}^{*}l_{n}^{\left(a\right)}}+\frac{6\tau }{\tau_{n}^{\left(0\right)}{l_{n}^{*}}^{2}}}$$ (7)

where for each layer, $\tau_{n}^{\left(0\right)}=(k_{n}^{2}D_{Bn}{)}^{-1}$ is the correlation time for a single scattering event. Here, *k*_*n*_ is the wavenumber of the light at wavelength *λ* and *D*_*Bn*_ is the Brownian diffusion coefficient for the n-th layer, a parameter that describes the scatterer dynamics inside each layer and thus can quantify the motion of RBC in thick/deep tissue [2, 11]. The field autocorrelation at the surface, *G*_0_(***r***, *τ*), can be obtained by solving Eq (6) in the Fourier domain with respect to the transverse coordinate ***ρ*** as

$$\hat{G}(q,z,\tau )=\int d^{2}\rho G(r,\tau )exp\;(iq\rho )$$ (8)

where ***q*** is the radial spatial frequency. By applying the boundary conditions as described in Li et al., the Fourier transform of the field autocorrelation function of diffuse reflected light, *G*_0_(***r***, *τ*), measured at the surface can be written as

$${\hat{G}}_{0}(q,z=0,\tau )=\frac{\mathrm{numerator}}{\mathrm{denominator}}$$ (9)

In this work, the human head was modeled as a two-layer medium, as shown in Fig 1. The first layer accounts for skull as well as the static (i.e., no moving scatterers) scalp and the second layer has a semi-infinite geometry that accounts for the brain cortex. For n = 2, d_2_ → ∞: hence, the numerator and denominator for ${\hat{G}}_{0}(q,z=0,\tau )$ can be written as

$$numerator=s_{0}z_{0}\{\beta_{1}D_{1}\;\cosh[\beta_{1}(d_{1}‐z^{\prime })]+\beta_{2}D_{2}\;\sinh[\beta_{1}(d_{1}‐z^{\prime })]\}$$ (10)

$$denominator=\beta_{1}(D_{1}+\beta_{2}D_{2}z_{0})\cosh(\beta_{1}d_{1})+(\beta_{2}D_{2}+\beta_{1}^{2}D_{1}z_{0})\sinh(\beta_{1}d_{1})$$ (11)

where $z_{0}^{\prime }=1/{\mu_{s}^{\prime }}^{\left(1\right)}$, and for each layer, $\beta_{n}(q,\tau )=[\alpha_{n}^{2}(\tau )+q^{2}{]}^{1/2},\;D_{n}=cl_{n}^{*}/3$ is the photon diffusion coefficient, and c is the speed of light.

For n = 1, a homogenous layer, the numerator and denominator for Eq (9) are

$$numerator=s_{0}z_{0}exp(-\beta_{1}z^{\prime })$$ (12)

$$denominator={1+\beta }_{1}z_{0}$$ (13)

Lastly, by performing the inverse Fourier transform of Eq (9) with respect to ***q***, the autocorrelation function for the electric field measured at the surface can be obtained as

$$\begin{array}{l} G_{0}(r,\tau )=\frac{1}{{\left(2\pi \right)}^{2}}\int d^{2}q\;{\hat{G}}_{0}(q,z=0,\tau )\;\exp\left(-iq\rho \right)\\ \;=\frac{1}{2\pi }\int dq\;{\hat{G}}_{0}(q,z=0,\tau )qJ_{0}(\rho q) \end{array}$$ (14)

where *J*_0_ is the zero-order Bessel function of the first kind.

### DCS instrumentation and data analysis

**Fig 2:**
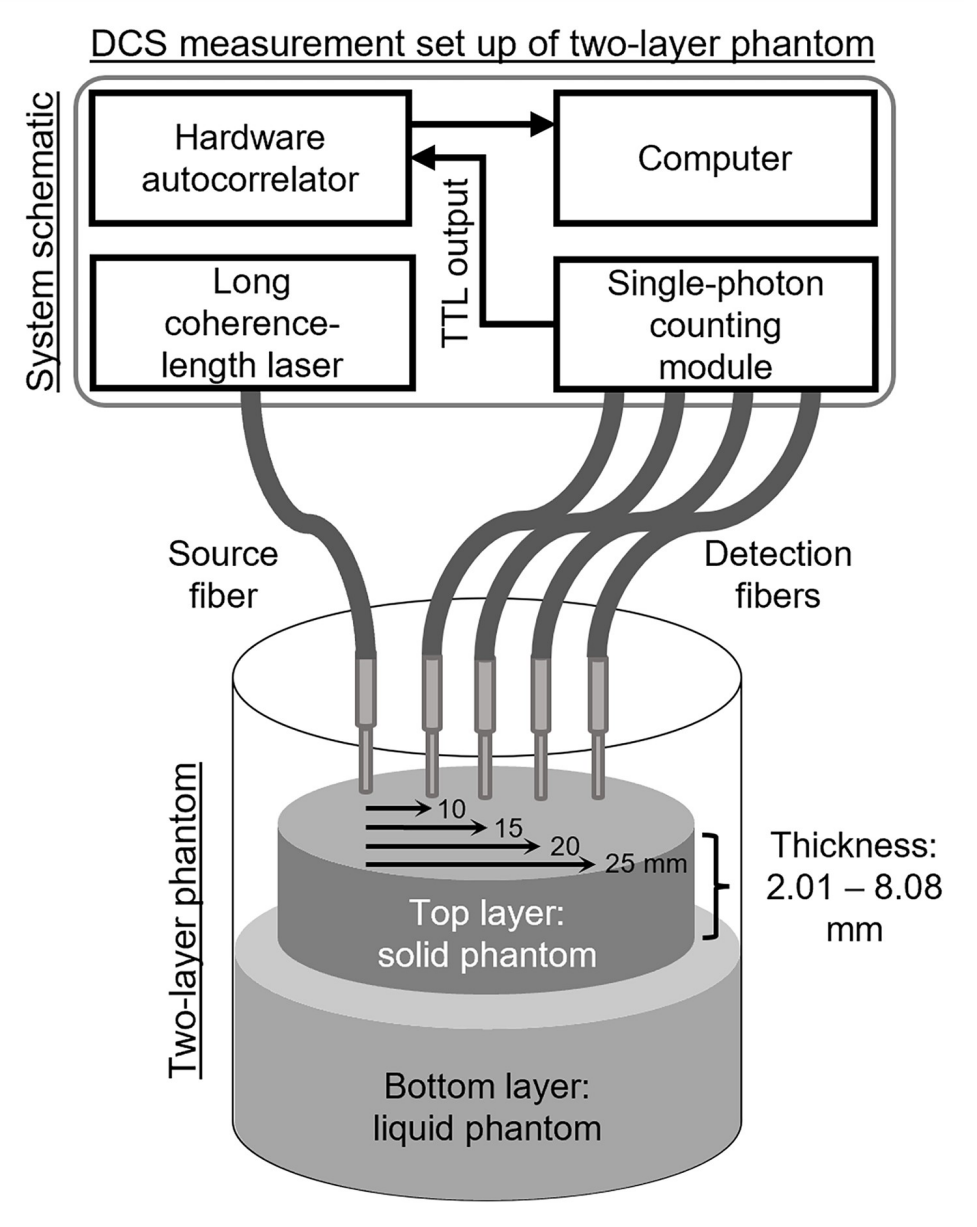
Schematic of the custom DCS system and setup for the two-layer phantom experiment with a single source fiber and four detection fibers at 10 mm, 15 mm, 20 mm and 25 mm from the source fiber. The top and bottom layers are solid and liquid phantom layers respectively.

A custom built DCS system was used in this work. Fig 2 presents a simple schematic of the DCS system. Briefly, a single long-coherence length laser at wavelength λ = 785 nm (DL785-70-3O, CrystaLaser, Reno, NV) was used for illumination through a multi-mode fiber (core dimeter 400 μm, N.A. = 0.39). On the detection side, four single-mode fibers (core dimeter 3.5 μm, N.A. = 0.13), at four different SD distances (SD1 = 10 mm, SD2 = 15 mm, SD3 = 20 mm, SD4 = 25 mm) from the source fiber, were led into a single-photon counting module (SPCM-AQ4C, Excellitas, Canada) comprised of 4 individual detectors. Following that, an 8-channel hardware correlator (Flex05-8ch, Correlator.com, NJ), with 4 channels being available for each SD distance, was used to auto-correlate the detected intensities with the sampling frequency of 2 Hz, from which the intensity autocorrelation (*g*_2_) was obtained. Time span for a single measurement was 100 s. Each phantom measurement was repeated ten times, between which the probe was taken off the phantom and put back in place. The photon count rate wasn’t adjusted by tuning the laser power during the measurement.

During data processing, the autocorrelation correction factor, *β*, was estimated from the first few data points (τ = 0.5×10^−6^ s to τ = 0.8×10^−6^ s) of the average of measured *g*_2_ over time. The averaged *g*_2_ was then converted to *g*_1_ using Eq (5). Additionally, the SNR for each delay time over the 100 s measurement was calculated using *SNR* = (*g*_2_(*τ*)−1)/*σ*(*g*_2_(*τ*)) [17]. For the two-layer model, the *g*_1_ curve from τ = 1×10^−6^ s to τ = 3×10^−3^ s for each measurement was then fitted to the solution of the correlation diffusion equation using Eq (14) with n = 2 to extract top layer thickness (*d*_1_) and bottom layer Brownian diffusion coefficient (*D*_*B*2_) [2]. In addition, ground-truth *D*_*B*2_ was extracted using Eq (14) with n = 1 on the bottom-layer-only (homogenous) measurements. For comparison purpose, the *g*_1_ curve above 0.7 was fitted to the conventional single-layer model (Eq (2)) to extract the Brownian diffusion coefficient times *α* (*αD*_*B*_) [32].

For both one-parameter single-layer models–Eq (2) and Eq (14) with n = 1 –we used MATLAB (Mathworks, Inc., Natick, MA) “fminsearch” function to perform least-square fitting. For the two-parameter two-layer model (Eq (14) with n = 2), MATLAB “fmincon” function was used with SNR as a weighting function for each delay time. The weighting was implemented to reduce noise on the fitted curve. The lower and upper bound for *d*_1_ and *D*_*B*2_ were set to be in the range of 0 to 20 mm and 0 to 10^−6^ cm^2^/s respectively with the initialization set to 0 for both parameters. The *D*_*B*1_ was set to zero for the use of a solid and thus a static layer [22]. The photon diffusion coefficients, *D*_1_ and *D*_2_, were calculated using measured optical properties and were held constant. These assumptions reduced the free parameters only to *d*_1_ and *D*_*B*2_ during fitting. The average and standard deviation from the 10 repeated measurements were calculated for fitted *d*_1_ and fitted *D*_*B*2_.

To confirm the fitting quality of the two-layer model, we computed the residual sum of squares (RSS = ∑[*g*_1,fitted_(*τ*)−*g*_1,measured_(*τ*)]^2^) between the fitted and measured *g*_1_ and generated the corresponding contour plots with various *d*_1_ and *D*_*B*2_ values. In addition, if the global minimum of RSS(*d*_1_, *D*_*B*2_) indicates a different *d*_1_ and *D*_*B*2_ pair than the fitted values by MATLAB “fmincon”, the *d*_1_ and *D*_*B*2_ pair from the contour plot would be used as the fitted values, because “fmincon” does not guarantee convergence to the global minimum.

To test how the uncertainty in optical properties will affect the two-layer model (Eq (14) with n = 2), we modified each of the *μ*_*a*_ and $\mu_{s}^{\prime }$ of each layer used in the fitting by ± 20%, and then performed the same two-parameter fitting procedure as described previously. Percent changes of the fitted *d*_1_ from true *d*_1_ were computed by 100%⋅(*d*_1,fitted_−*d*_1,true_)/*d*_1,true_; percent changes of the fitted *D*_*B*2_ from true *D*_*B*2_ (fitted by the true *μ*_*a*_ and $\mu_{s}^{\prime }$ using the Eq (14) with n = 1) were computed by 100%⋅(*D*_*B*2,fitted_−*D*_*B*2,true_)/*D*_*B*2,true_.

### Two-layer phantom experiment

The top layer of the two-layer optical phantoms, as shown in Fig 2, was a solid, static phantom. The solid phantom was made using silicone: 800 mL of part A, 80 mL of part B from SORTA-clear 40 (Smooth-on, Macungie, PA), and 240 mL of silicone thinner (Smooth-on, Macungie, PA). To mimic tissue *μ*_*a*_ and $\mu_{s}^{\prime }$, 1.5 g of titanium dioxide (Atlantic Equipment Engineers, Upper Saddle River, NJ) and 400 μL of Higgins black India ink (Chartpak, Inc., Leeds, MA) were added to the silicone base, mixed with a handheld electrical mixer, and then poured onto a petri dish. This recipe follows the standard mixing ratios as reported in the literature [33, 34]. The thickness of the mixture inside the petri dish was varied to mimic different skull thicknesses. Following that, the mixture was degassed for ~1 hour in a vacuum chamber to remove air bubbles and left to solidify for ~24 hours. Lastly, the solid phantom was peeled out of the petri dish and the thickness (referred to as true thickness in later sections) was measured using a slide caliper. Human skull thickness varies with sex, age, etc., but it was reported to be within 1–10 mm [35, 36]. As a result, in this work, seven different thickness values ranging from 2.01 mm to 8.08 mm were tested (P1 = 2.01, P2 = 3.28, P3 = 3.87, P4 = 4.65, P5 = 4.99, P6 = 5.96, and P7 = 8.08 mm) for the top layer.

The bottom layer in the two-layer phantoms was a liquid phantom which represented the human brain cortex, as shown in Fig 2. The phantom was made with 300 mL of milk at room temperature (2% reduced, Giant Eagle, Pittsburgh, PA), 400 mL of water, and 25 μL of Higgins black India ink (Chartpak, Inc., Leeds, MA). The India ink was used as the absorber and the milk was used for mimicking moving scatterers in the tissue [33]. Apart from the two-layer phantoms, no first layer added to the liquid phantom, referred to as P0, corresponds to a homogenous phantom, which in later sections will be referred to as the single-layer phantom.

Because the top layer solid phantoms were too thin for accurate measurements of optical properties with diffuse imaging techniques, thicker homogeneous phantoms were also made from the same batch of material. These top layer phantoms were cylindrical in shape, with a diameter of 8.2 cm and a height of 5.4 cm. For bottom layer, the same liquid phantom was used for measurement of optical properties as used in the two-layer phantom.

The optical properties of the both solid and liquid phantoms were measured at 690 nm and 830 nm using a frequency-domain NIRS system (OxiplexTS, ISS, Champaign, IL) and then converted to the DCS operating wavelength of 785 nm. First, ink absorption was assumed to be spectrally flat, hence the *μ*_*a*_ at 690 nm and 830 nm from NIRS were averaged to obtain *μ*_*a*_ at 785 nm for DCS. On the other hand, the $\mu_{s}^{\prime }$ at 690 nm and 830 nm were fitted to a power law to extract $\mu_{s}^{\prime }$ at 785 nm [37]. These optical properties’ values (top layer: *μ*_*a*_ = 0.11 cm^−1^ and $\mu_{s}^{\prime }$ = 8.50 cm^−1^, bottom layer: *μ*_*a*_ = 0.12 cm^−1^ and $\mu_{s}^{\prime }$ = 10.46 cm^−1^) were used to obtain *D*_n_ during fitting of *g*_1_, and will be referred to as baseline optical properties in later sections. The optical properties’ values for each layer were close to the range for human skull and brain tissue as reported in the literature (skull: *μ*_*a*_ = 0.21–0.36 cm^−1^ and $\mu_{s}^{\prime }$ = 11.90–7.70 cm^−1^ for λ = 674–956 nm, brain tissue: *μ*_*a*_ = 0.17–0.21 cm^−1^ and $\mu_{s}^{\prime }$ = 8–11.20 cm^−1^ for λ = 674–956 nm) [38, 39].

During measurement, a solid phantom was placed on the liquid homogeneous phantom inside a glass beaker to mimic a two-layer geometry, as shown in Fig 2. The solid top layer was suspended by a wire scaffold (not shown in Fig 2) such that it was just touching the surface of the liquid layer at the bottom. The source and detector fibers were placed in contact with the top layer with the fibers being perpendicular to the surface. A black cloth was used to cover the beaker, blocking ambient light during measurement acquisition.

## Results

### Influence of top layer thickness on correlation curves

**Fig 3:**
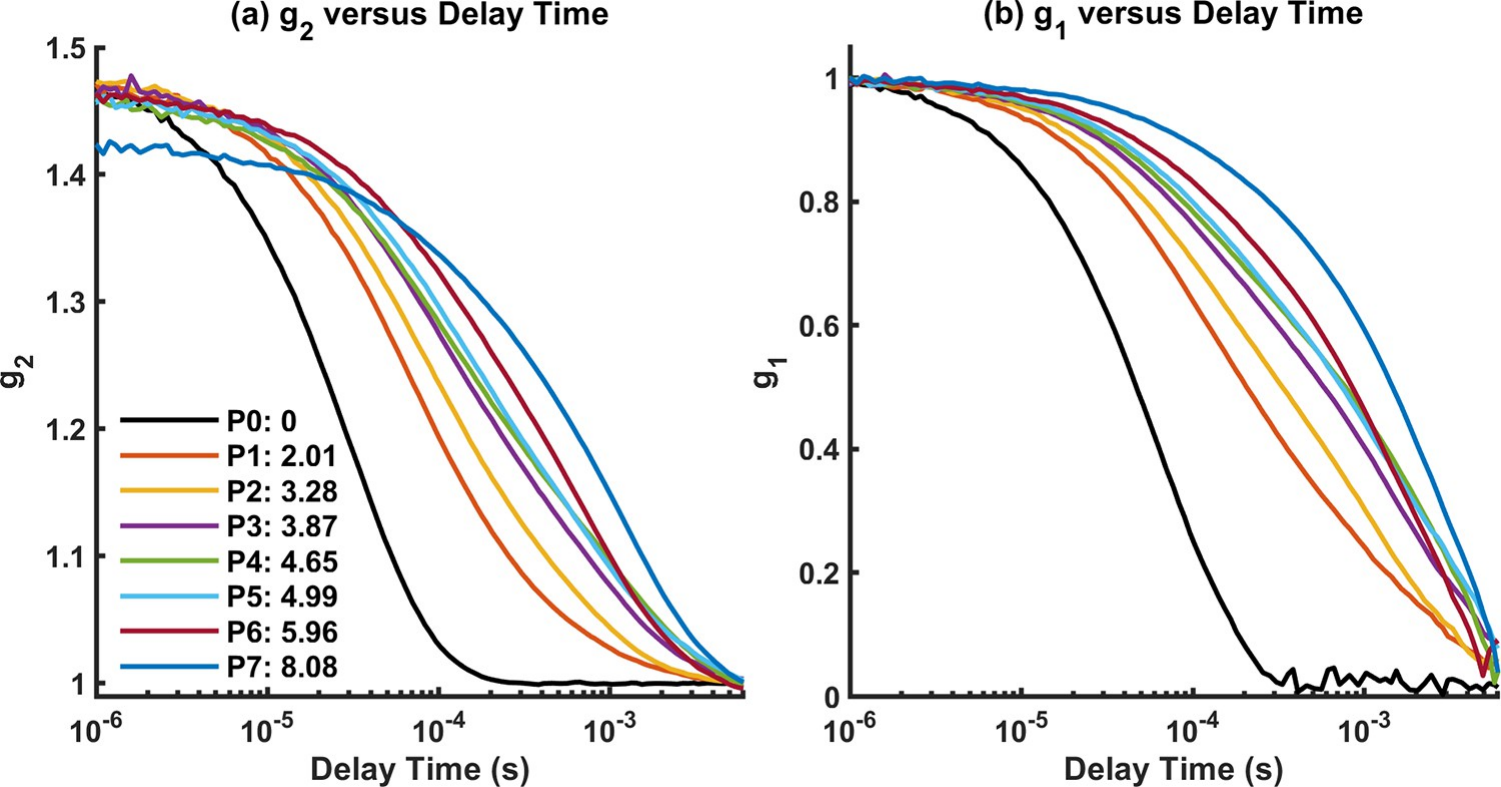
(a) *g*_2_ from homogenous phantom P0 to two-layer phantom P7, where *d*_1_ is the thickness of the top layer phantom, shown for SD = 20 mm. (b) Corresponding *g*_1_ curves calculated from *g*_2_ in (a).

**Fig 4:**
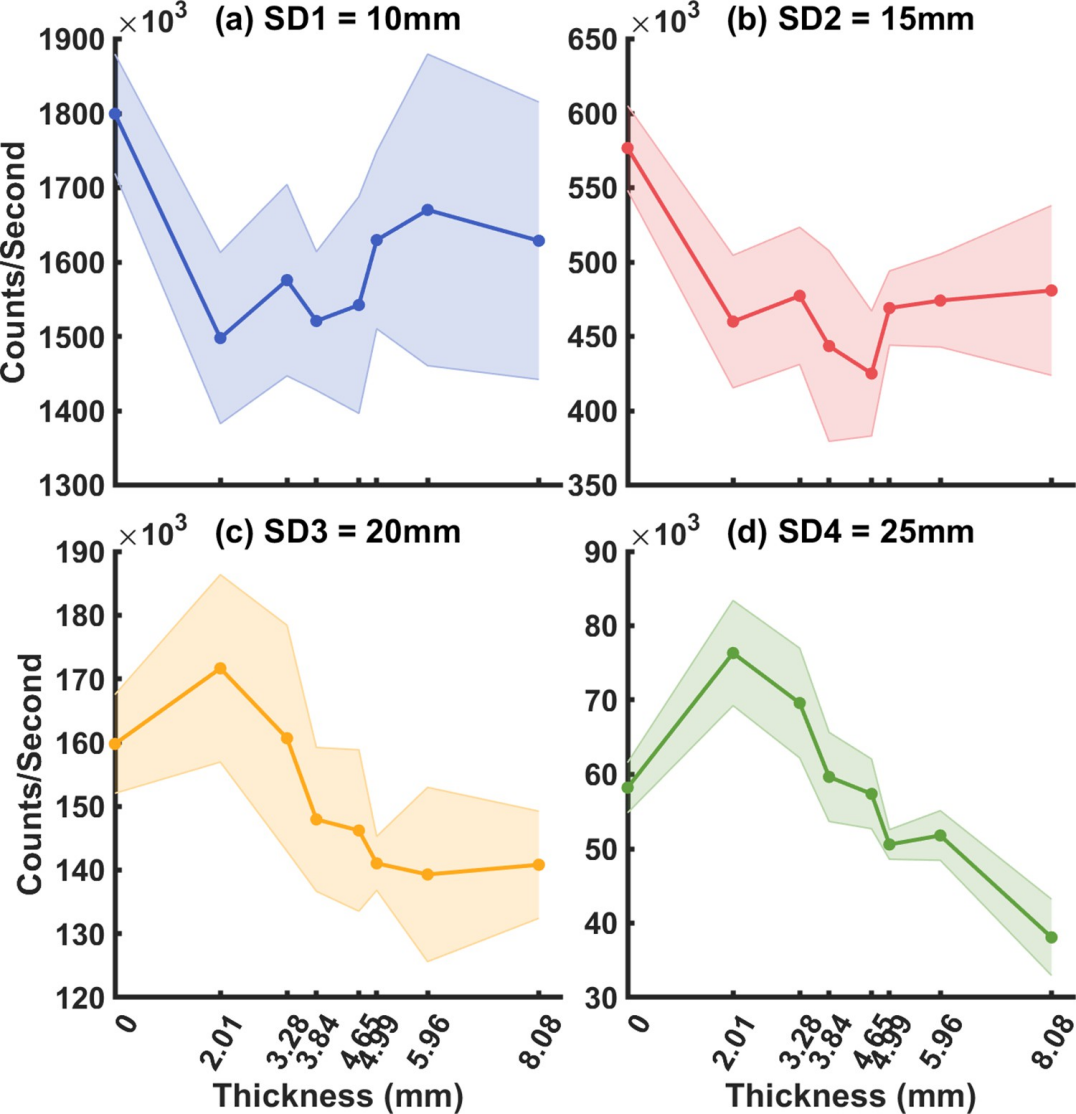
(a-d) Photon count rate vs. top layer thickness (P0-P7) for SD1-SD4 respectively.

The two-layer phantoms were measured with DCS, where the bottom layer liquid phantom stayed the same, but the top layer solid phantom was varied between different thicknesses, referred to as P0-P7 (P0 = no first layer, P1 = 2.01 mm, P2 = 3.28 mm, P3 = 3.87 mm, P4 = 4.65 mm, and P5 = 4.99 mm, P6 = 5.96 mm, P7 = 8.08 mm). Fig 3A shows a single trial of the measured *g*_1_ (averaged over 100s) for all phantoms at a SD3 = 20 mm. Fig 3B shows the corresponding *g*_1_. In addition, photon count rates for each SD (SD1 = 10 mm, SD2 = 15 mm, SD3 = 20 mm, SD4 = 25 mm) across P0-P7 are shown in Fig 4A–4D. We can see that both *g*_2_ and *g*_1_ curves have distinct shape changes with various top layer thickness. This observation thus works as the basis for the feasibility of extracting the top layer thickness.

### Two-layer vs. the single-layer model

**Fig 5:**
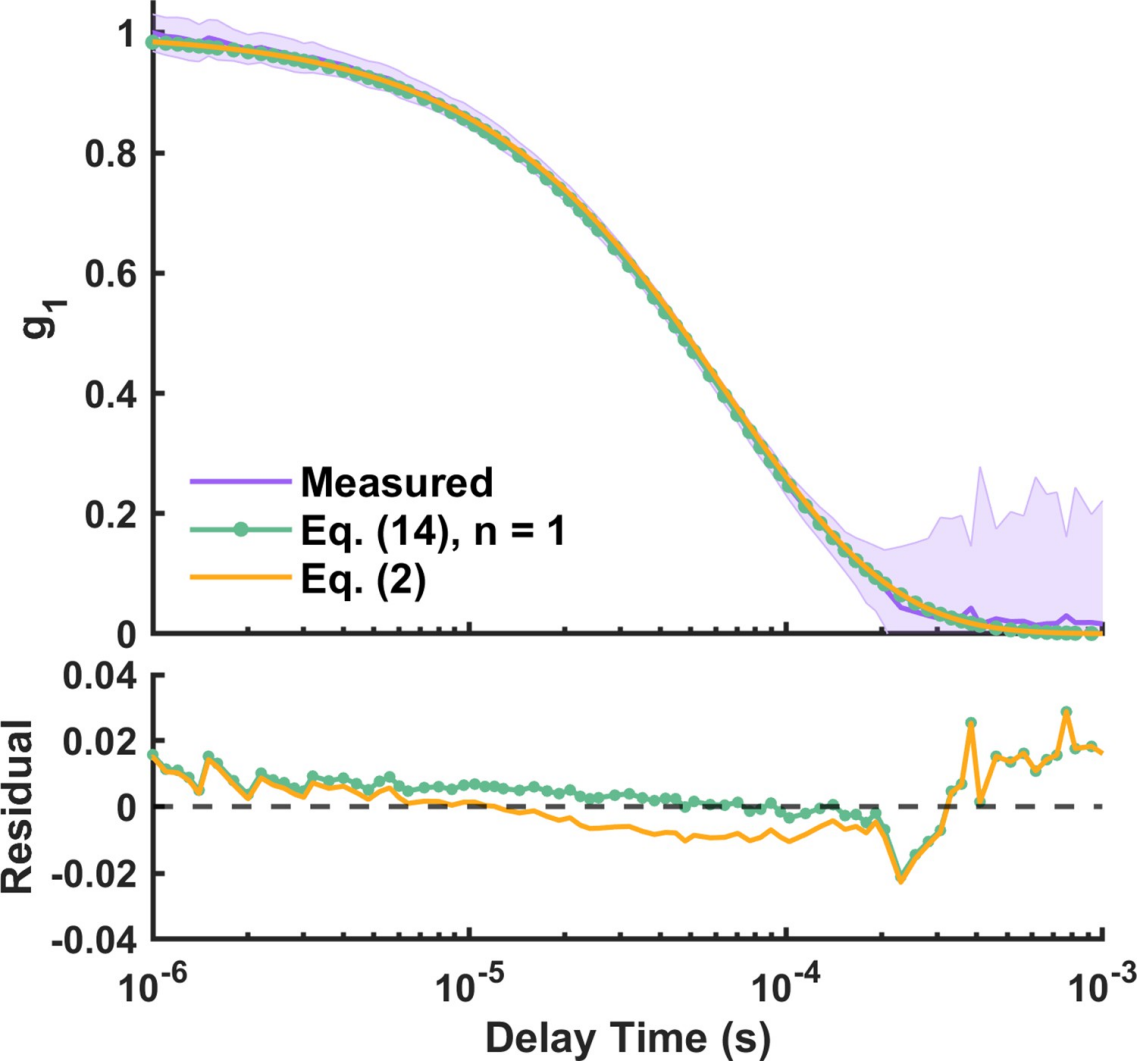
Measured and fitted *g*_1_ at SD3 (20 mm) for homogenous phantoms P0. Purple: measured *g*_1_ with standard deviation. Green with circle: Eq (14) with n = 1. Orange: conventional single-layer model (Eq (2)).

For the homogenous phantom P0, Eq (14) with n = 1 was used to extract the ground-truth *D*_*B*2_; Eq (2) was used to extract the *αD*_*B*_. Fig 5 shows an example of the measured and fitted *g*_1_ from a single trial of measurement for P0 at SD3 = 20 mm. The purple line and shaded region stem from the average and standard deviation of *g*_1_ over 100 s per delay time. The green line with circle and orange line represents fitted *g*_1_ using Eq (14) with n = 1 and Eq (2) respectively. The two homogenous fits (green and yellow lines) closely match each other, with the residual (measured *g*_1_ minus fitted *g*_1_) mostly centered around zero.

**Fig 6:**
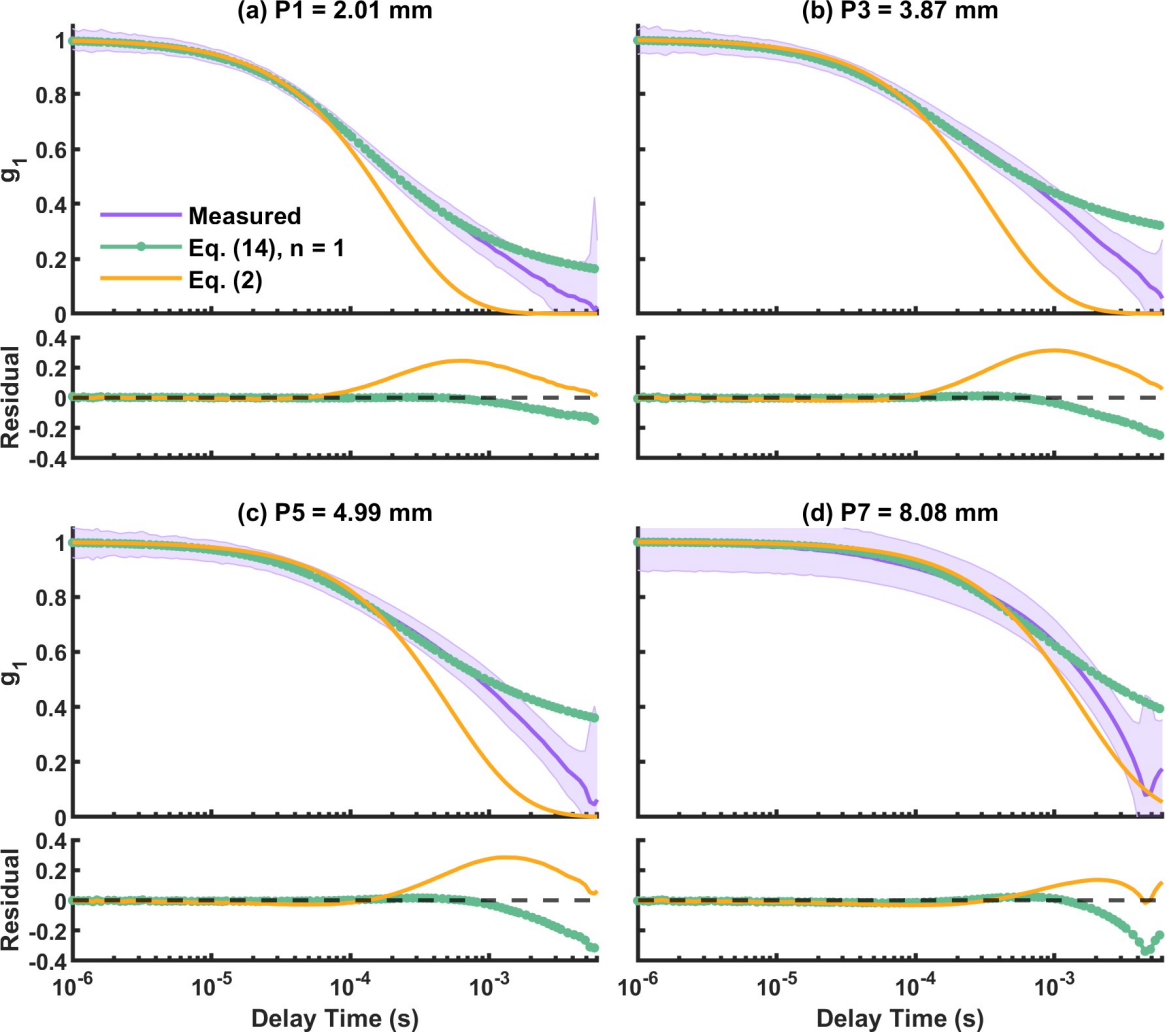
(a-d) Measured and fitted *g*_1_ at SD3 (20 mm) for two-layer phantoms P1 (2.01 mm), P3 (3.87 mm), P5 (4.99 mm), P7 (8.08 mm). Purple: measured *g*_1_ with standard deviation. Green with circle: two-layer model. Orange: single-layer model (Eq (2)).

The measured *g*_1_ curves from two-layer phantoms P1 to P7 were fitted to the two-layer analytical model to extract *d*_1_ and bottom layer flow (*D*_*B*2_), and the conventional single-layer analytical model (Eq (2)) to extract single-layer flow (*αD*_*B*_). Fig 6A–6D shows examples of the measured and fitted *g*_1_ for the two-layer phantoms P1 = 2.01 mm, P3 = 3.87 mm, P5 = 4.99 mm, and P7 = 8.08 mm at SD3 = 20 mm. The purple solid lines with shaded regions represent the mean and standard deviation of the measured *g*_1_; the green lines with circle and orange lines represent the fitted *g*_1_ using two-layer and single-layer model (Eq (2)) respectively. We can see that although the fitted *g*_1_ by the two-layer model did not describe the entire measured *g*_1_ accurately, it still performed better than the one-layer model by its ability to change the slope with different *d*_1_. This is further quantified in terms of residuals as seen in Fig 6, where the two-layer fit shows smaller residuals at longer delay times compared to the single layer fit.

### Simultaneous fitting for thickness and flow using a two-layer model

**Fig 7:**
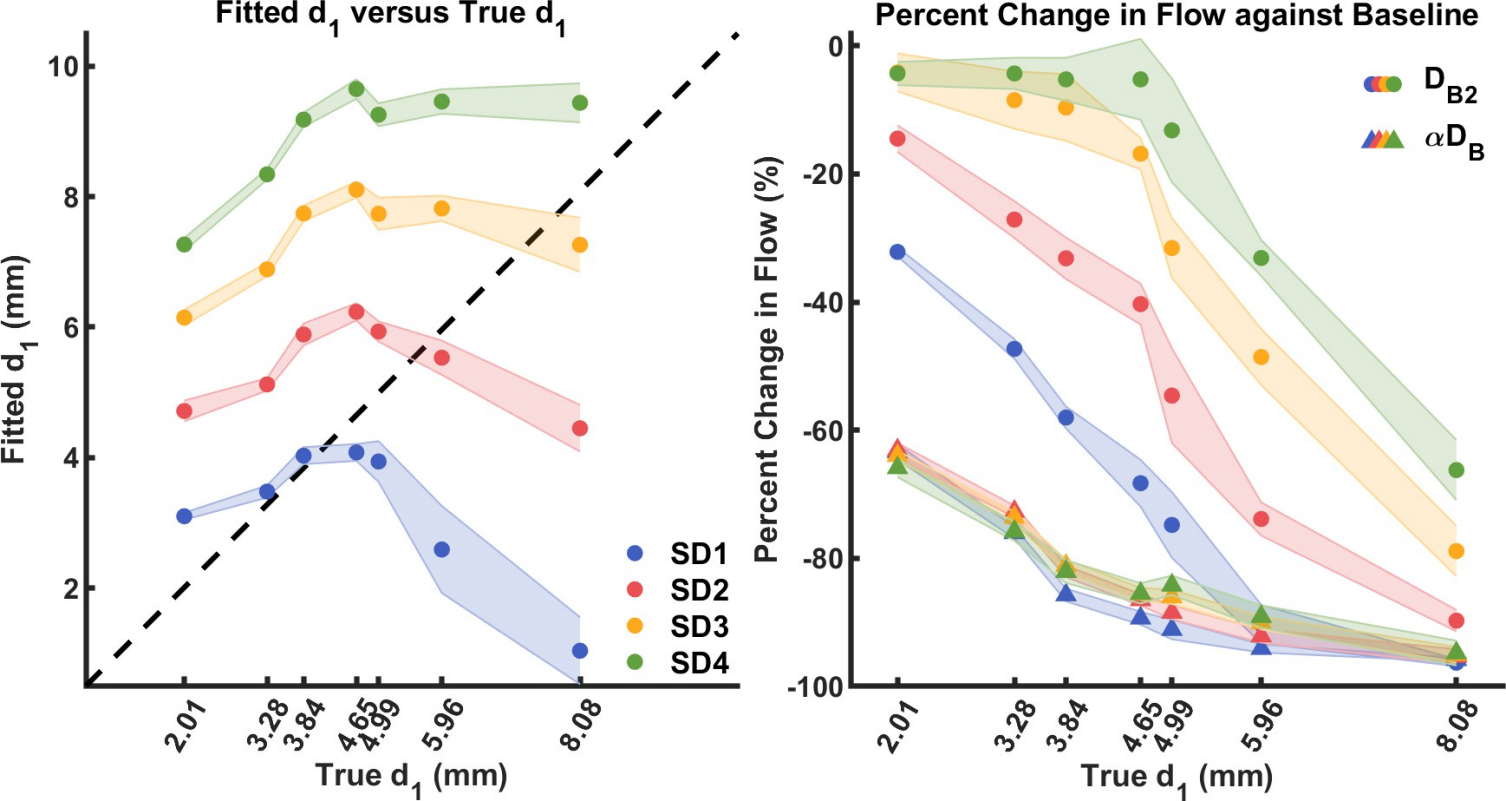
(a) Comparison between fitted and true *d*_1_ for various top layer thicknesses at each SD distance. Bule: SD1 = 10 mm. Red: SD2 = 15 mm. Orange: SD3 = 20 mm. Green: SD4 = 25 mm. Mean and standard deviation of fitted *d*_1_ were derived from ten repeated measurements. (b) Percent differences between layered phantom (P1-P7) and P0 in *αD*_*B*_ using the single-layer model (triangle), and *D*_*B*2_ using the two-layer model (circle) vs. true *d*_1_ values. Mean and standard deviation of fitted flow were derived from ten repeated measurements.

Two parameters–top layer thickness (*d*_1_) and bottom layer Brownian diffusion coefficient (*D*_*B*2_)–were extracted by fitting the two-layer model to the measured *g*_1_, while only the Brownian diffusion coefficient times *α* (*αD*_*B*_) was extracted by the conventional single-layer model (Eq (2)). The S1 Table summarizes the results of these fitted *d*_1_, *D*_*B*2_ and *αD*_*B*_ value for P0-P7 phantoms, where the average and standard deviation were derived from the ten repeated measurements. For visualization, Fig 7A shows correlations between the true and fitted *d*_1_ at each SD distances for P1-P7. Linear fitting (MATLAB “fitlm”) between fitted and true *d*_1_ for all P1-P7 did not show a significant correlation. However, we found a significant positive linear correlation for all SD distances when fitting up to P5 = 4.99 mm (SD1 = 10 mm: p = 0.043 < 0.050, SD2 = 15 mm: p = 0.033 < 0.050, SD3 = 20 mm: p = 0.028 < 0.050, SD4 = 25 mm: p = 0.022 < 0.050). We observed that the fitted *d*_1_ values at larger SD distances were overestimated more, but they follow a linear trend when *d*_1_ < 5 mm. This could indicate that for a set *d*_1_, the fitting accuracy depends on the SD distance and top layer thickness.

Since the two-layer fitting yielded *D*_*B*2_ and the single-layer fit *αD*_*B*_, a direct comparison between the values was not possible. To compare the two models, we thus obtained the ground truth *D*_*B*2_ and *αD*_*B*_ from measurements on P0 (homogeneous liquid phantom that provides the most accurate prediction of flow), and then calculated the percent differences between values extracted from each layered phantom (P1-P7) to their ground truths. For P0, the ground truth *D*_*B*2_ and *αD*_*B*_ were calculated using single-layer models Eq (14) with n = 1 and Eq (2) respectively.

As shown in Fig 7B, for the single-layer model (triangles), fitted *αD*_*B*_ at all SD distances quickly deviates from the baseline flow of P0 as *d*_1_ increases (around 65% difference at the thinnest P1 = 2.01 mm), confirming its inaccuracy for flow estimation at non-negligible top layer thicknesses. On the other hand, from the results of the two-layer model (circles), we observed that the estimation of *D*_*B*2_ changes with SD distances: At shorter SD distances (SD1 = 10 mm and SD2 = 15 mm) fitted *D*_*B*2_ decreases in a similar way to the single-layer model but with smaller discrepancies compared to the reference. At longer SD distances, however, fitted *D*_*B*2_ only decreases to less than or around 15% from baseline until *d*_1_ = 4.65 mm for SD3 = 20 mm and *d*_1_ = 4.99 mm for SD4 = 25 mm. For even the thickness P7 = 8.08 mm, *D*_*B*2_ estimated from SD4 decrease to around 65% from baseline, which is a similar discrepancy to single-layer model at the thinnest P0 = 2.01 mm. Longer SD distances (yellow and green circles in Fig 7B for SD3 and SD4 respectively), which are more sensitive to the bottom layer, did provide better fit for *D*_*B*2_ with the two-layer model. These findings suggest that the two-layer model can estimate the bottom layer flow more accurately than the single-layer model when a top static layer is present.

**Fig 8:**
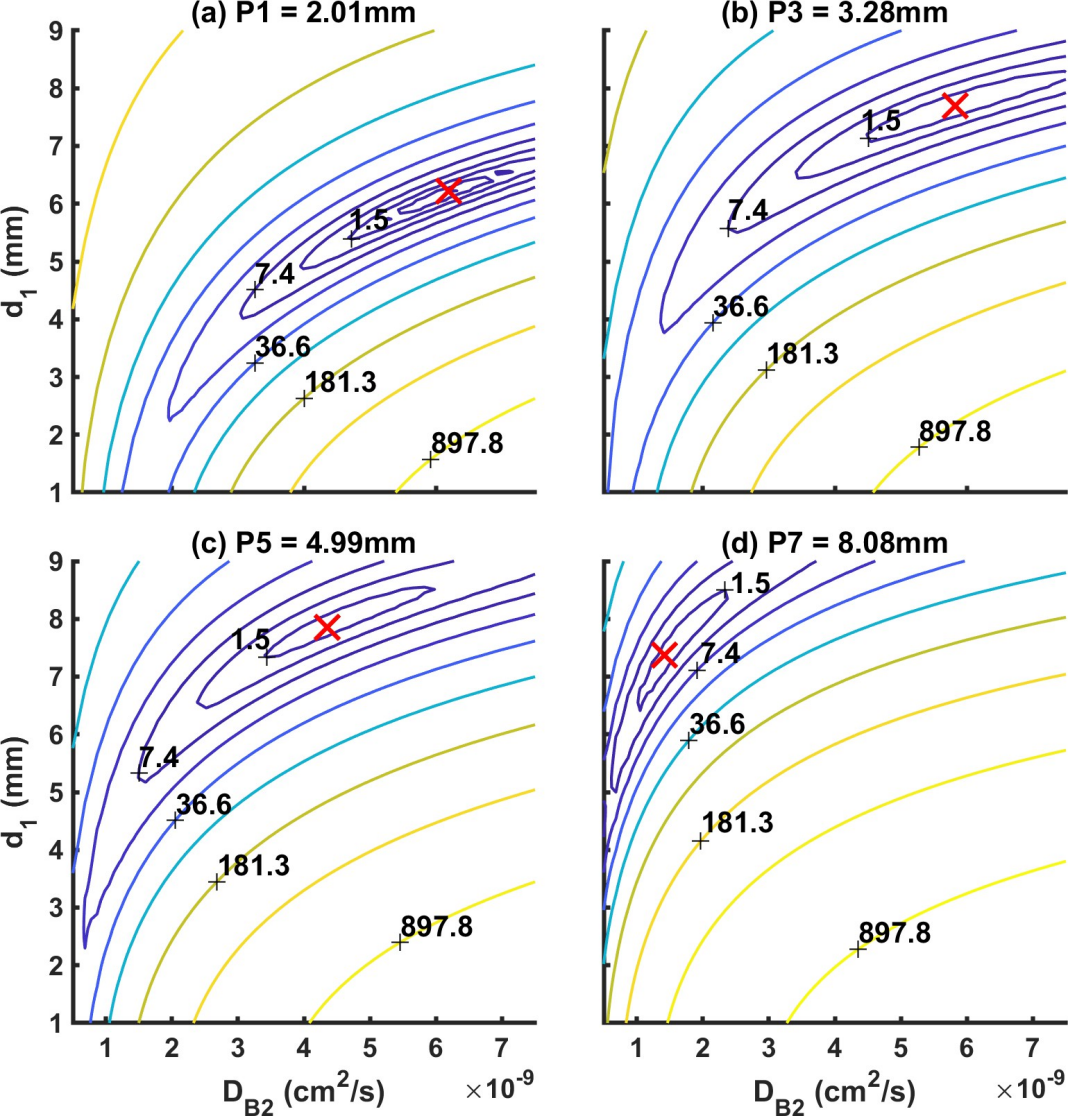
RSS between the fitted and measured *g*_1_ by varying *d*_1_ from 1 mm to 9 mm, and *D*_*B*2_ from 0.5×10^−9^ cm^2^/s to 7.5×10^−9^ cm^2^/s. (a-d) Example RSS of one out of ten repeated measurements for P1 = 2.01 mm, P3 = 3.28 mm, P5 = 4.99 mm, and P7 = 8.08 mm respectively at SD3 = 20 mm. Red cross: Global minimum.

To further demonstrate the feasibility of simultaneously extracting the top layer thickness and flow and the fitting quality, the contour plots of RSS (residual sum of squares) between the measured and fitted *g*_1_ were generated by varying the values of *d*_1_ and *D*_*B*2_ pair. Fig 8A–8D shows the example RSS for P1 (2.01 mm), P3 (3.28 mm), P5 (4.99 mm), and P7 (8.08 mm) at SD3 (20 mm). We can see that RSS in all cases converged to a single minimum as shown by the red cross. As discussed in the previous session, the accuracy of fitted *d*_1_ and *D*_*B*2_ depend on the SD distance. Thus, examples in Fig 8A–8D aim to show the convergence of RSS instead of the fitting accuracy. The convergent patterns of RSS suggest that it is possible to fit for of *d*_1_ and *D*_*B*2_ simultaneously.

### Model sensitivity on layer optical properties

The two-layer model assumes optical properties of the top and bottom layers during fitting of *d*_1_ and *D*_*B*2_. Precise knowledge of the optical properties in each layer, however, can be hard to obtain for some applications [40]. To evaluate the influence of layer optical properties on the reconstruction of *d*_1_ and *D*_*B*2_, we separately varied the *μ*_*a*_ and $\mu_{s}^{\prime }$ in each layer by ± 20% and performed the same two-parameter fitting procedure as described previously. The fitted *d*_1_ and *D*_*B*2_ were then compared to their expected values by 100%⋅(*d*_1,fitted_−*d*_1,true_)/*d*_1,true_ and 100%⋅(*D*_*B*2,fitted_−*D*_*B*2,true_)/*D*_*B*2,true_ respectively. The ground truth *D*_*B*2_ were fitted by the baseline *μ*_*a*_ and $\mu_{s}^{\prime }$ using the Eq (14) with n = 1 from P0 (liquid layer only, no top layer).

**Fig 9:**
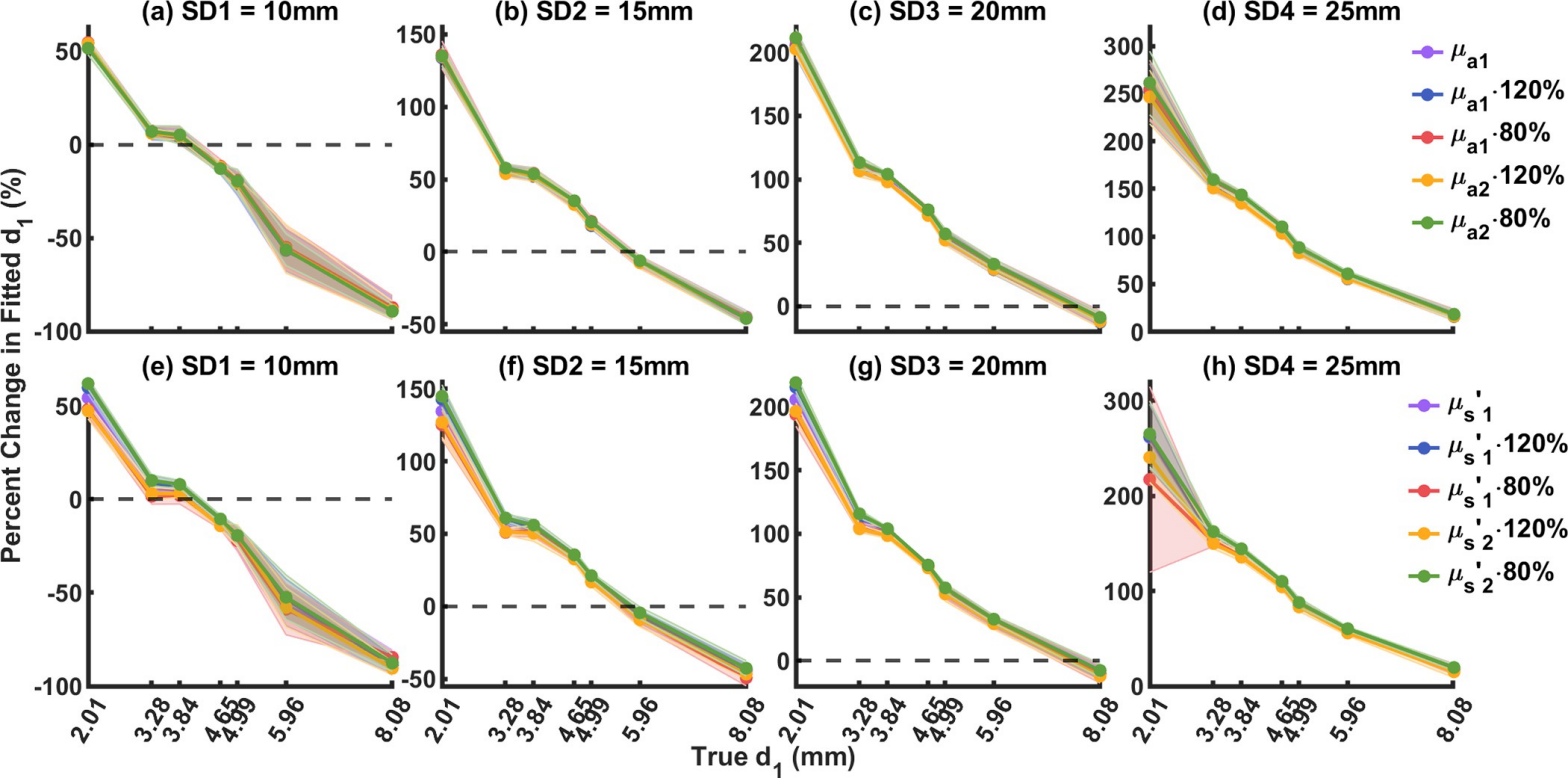
Influence of uncertainties in layer optical properties on fitted *d*_1_. Purple: baseline. Blue: first layer increased by 20%. Red: first layer decreased by 20%. Yellow: second layer increased by 20%. Green: second layer decreased by 20%. (a-d) Influence of layer *μ*_*a*_, SD1-SD4. (e-h) Influence of layer $\mu_{s}^{\prime }$, SD1-SD4.

For *d*_1_, Fig 9A–9D and 9E–9H show how changes in *μ*_*a*_ and $\mu_{s}^{\prime }$ affect the fitted *d*_1_ respectively at each top layer thickness from SD1 to SD4. The overlapping curves in the figure indicate that changes in *μ*_*a*_ and $\mu_{s}^{\prime }$ by ± 20% in each layer minimally affect the fitted *d*_1_.

**Fig 10:**
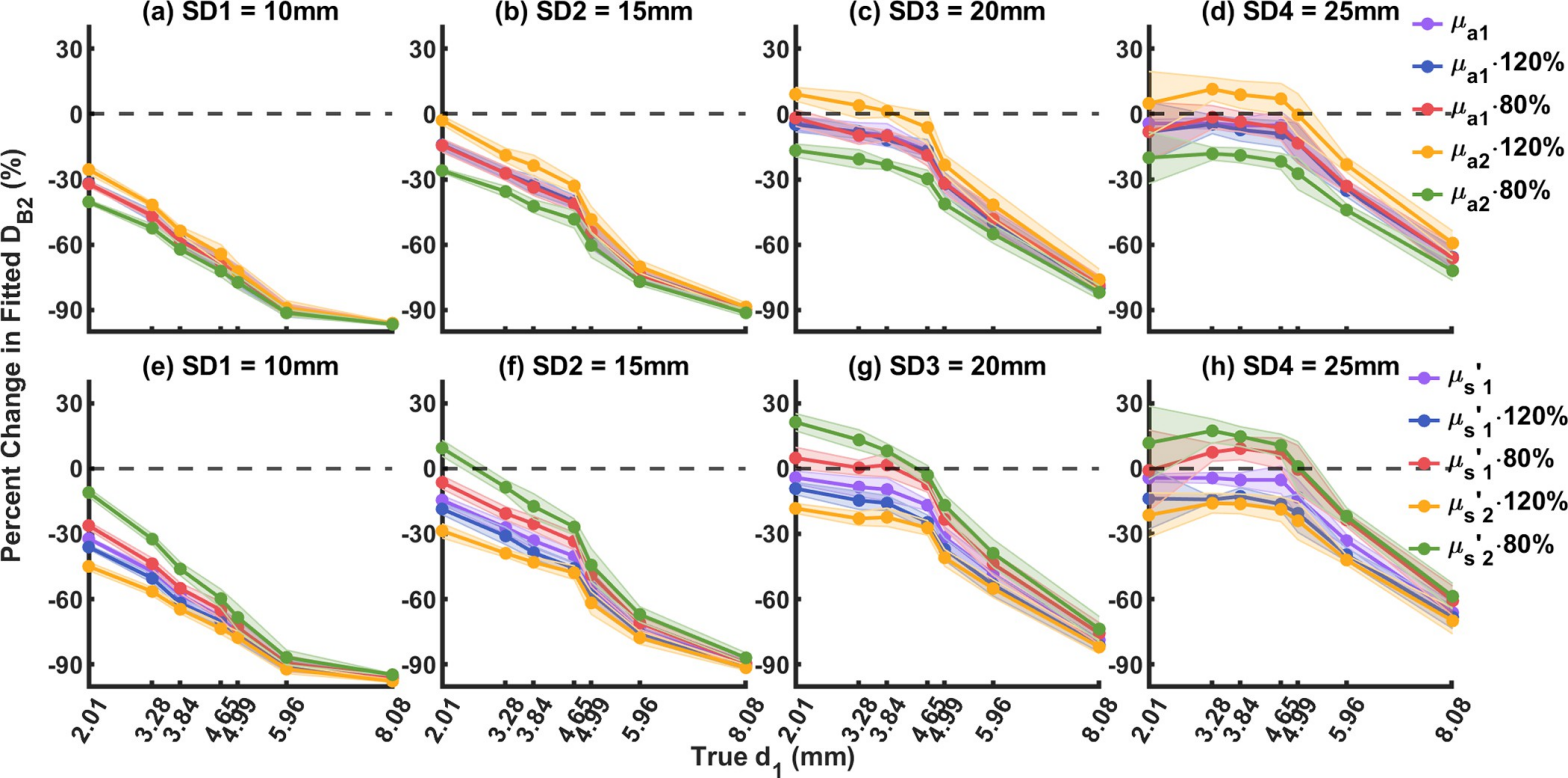
Influence of uncertainties in layer optical properties on fitted *D*_*B2*_. Purple: baseline. Blue: first layer increased by 20%. Red: first layer decreased by 20%. Yellow: second layer increased by 20%. Green: second layer decreased by 20%. (a-d) Influence of layer *μ*_*a*_, SD1-SD4. (e-h) Influence of layer $\mu_{s}^{\prime }$, SD1-SD4.

Fig 10A–10D shows how changes in *μ*_*a*_ affect the fitted *D*_*B*2_ at each top layer thickness from SD1 to SD4. We observed that changes in the first layer *μ*_*a*_ by ± 20% (red and blue) only minimally affect the fitted *D*_*B*2_. Changes in the second layer *μ*_*a*_, however, show that overestimation of *μ*_*a*_ (yellow) increases the fitted *D*_*B*2_ by around 15%; underestimation of *μ*_*a*_ (green) decreases the fitted *D*_*B*2_ by around 15%.

Fig 10E–10H shows how changes in $\mu_{s}^{\prime }$ by ± 20% affect the fitted *D*_*B*2_ at each top layer thickness from SD1 to SD4. We can see that for $\mu_{s}^{\prime }$ in both layers, overestimation of $\mu_{s}^{\prime }$ (blue and yellow) decreases the fitted *D*_*B*2_; underestimation of $\mu_{s}^{\prime }$ (red and green) increases the fitted *D*_*B*2_. In addition, changes in the second layer $\mu_{s}^{\prime }$ affect fitted *D*_*B*2_ more than the first layer $\mu_{s}^{\prime }$ (around 20% compared to around 10%).

Using the two-layer model, we found that the uncertainties in optical properties affect fitted *D*_*B*2_ more than fitted *d*_1_. Uncertainties in optical properties of ± 20% minimally affected the fitted *d*_1_, but those in each layer affected the fitted *D*_*B*2_ differently as discussed above.

## Discussion and conclusion

In this manuscript, a two-layer analytical model for CW-DCS adapted from Li et al. was adapted and validated for its feasibility in estimating layer thickness in addition to flow [22]. Two-layer phantoms were fabricated to mimic the brain geometry–with the top layer representing skull with finite thickness (up to ~8 mm) and the bottom layer representing brain cortex with semi-infinite geometry. DCS measurements were performed on phantoms with different thicknesses (from P0 = 0 mm to P7 = 8.08 mm). We found that, the shapes of *g*_2_ and *g*_1_ change with *d*_1_, indicating the possibility of extracting layer thickness beside flow from the DCS measurements. This finding was in compliance with Boas et al. who observed, while simulating burned tissue, a slower rate of decay in *g*_1_ as the thickness of the teflon layer increased resting above an intralipid liquid layer [11].

The two-layer model was then used to fit for *d*_1_ and *D*_*B*2_ simultaneously from the *g*_1_ curves. For a fixed two-layer phantom, the accuracy of fitted *d*_1_ and *D*_*B*2_ depends on the SD distance and the top layer thickness. For example, as shown in Fig 7A, fitted and true *d*_1_ showed significant positive linear relationship up to P5 = 4.99 mm. In addition, when the SD is not sensitive enough to the bottom layer, as shown in the case of SD1 = 10 mm for P7 = 8.08 mm, fitted *d*_1_ will largely deviate from its expected value. In another word, P7 = 8.08 mm is almost equivalent to a semi-infinite phantom to SD1 = 10 mm, causing inaccuracy in fitted *d*_1_ from the two-layer model.

For the flow in the bottom layer (*αD*_*B*_ and *D*_*B*2_), we first calculated their expected values by fitting the single-layer models (Eq (2) and Eq (14) with n = 1) to the measurements from the homogenous phantom P0 (no top layer). Then, as shown in Fig 7A for the conventional single-layer model, the percent difference between the fitted *αD*_*B*_ quickly deviates from its expected value when solid layers were added. On the other hand, as shown in Fig 7B, fitted *D*_*B*2_ from the two-layer model are more stable. Especially for the longer SD that are more sensitive to the bottom layer. The longest SD4 = 25 mm produced consistently accurate fitted *D*_*B*2_ (less than or around 10% difference from the expected value) for P1 = 2.01 mm to P5 = 4.99 mm. *D*_*B*2_ deviated more from the baseline at P6 = 5.96 mm and P7 = 8.08 mm, which we attribute partially due to low photon count rate as shown in Fig 4D.

To test the quality of two-parameter fitting, similar to Li et al. and Dong et al., contour plots of RSS between the fitted and measured *g*_1_ (Fig 8A–8D) were generated as a function of the fitted parameters *d*_1_ and *D*_*B*2_; the convergence to a single minimum demonstrated the feasibility of fitting *d*_1_ and *D*_*B*2_ simultaneously [22, 41].

Uncertainties in *μ*_*a*_ and $\mu_{s}^{\prime }$ were shown by Irwin et al. to have influence on the fitted blood flow using the conventional single-layer model [42]. To investigate how these uncertainties would influence the two-layer model, we varied the optical properties in each layer during the fitting by ± 20%. Our results (Figs 9 and 10) show that the fitted *d*_1_ was only mildly affected within ± 20% uncertainties. In addition, *μ*_*a*_ in the first layer only mildly affected the fitted *D*_*B*2_, but overestimated and underestimated *μ*_*a*_ in the second layer caused increased and decreased fitted *D*_*B*2_ respectively. On the other hand, overestimated and underestimated $\mu_{s}^{\prime }$ in both layers decreased and increased fitted *D*_*B*2_ respectively. This finding is consistent with that from Li et al., Zhao et al., and Irwin et al. [22, 27, 42].

It is to note that, there are a few limitations of the proposed model at the current setting. For example, although *d*_1_ and *D*_*B*2_ were fitted parameters, they were constrained to be within an expected range during fitting. If the first layer thickness is not within the expected range, the fitting results might be influenced. Further testing with even larger first layer thicknesses would shed light on this. Also, the accuracy of fitted *d*_1_ and *D*_*B*2_ was shown to be dependent on the SD distance or the sensitivity to each layer. Thus, investigation of incorporating multiple SD during the fitting could make fitting across a variety of layer thicknesses more robust. In addition, uncertainties in layer optical properties add inaccuracy to the fitted parameters. This could be overcome by measuring optical properties with frequency domain or time domain near-infrared spectroscopy [43–45].

Most importantly, the two-layer model proposed in this work is undoubtedly a simplified version of human head and neglected any flow dynamics or effects of optical properties in the scalp layer. This could potentially be overcome by implementing a three-layered model as shown by Wu et al. and Zhao et al. [25, 27]. However, a negligible scalp flow could also be achieved experimentally, as demonstrated by Baker et al., where an inflated cuff was placed around the head of the subject to eliminate scalp blood flow [46, 47]. In such a scenario, the model proposed in this work would be applicable due to the static first layer consisting of scalp and the skull.

In conclusion, over the past decade, CW-DCS has been established as a promising new technique for continuous brain monitoring and tracking brain perfusion changes [2, 13]. However, precise knowledge of extracerebral layers, such as skull, is also necessary and the reasons are twofold. First, if left uncorrected, the contribution from the skull can contaminate and underestimate cortical blood flow [28]. Second, the knowledge of continuous skull thickness could benefit detecting and monitoring skull deformation, something that is often found in patients with TBI [29]. To bridge the gap, this study adapted and validated a two-layer analytical model for CW-DCS that has the potential to sense skull thickness in addition to blood flow in the cortex layer in a continuous manner. Further studies will help improve model accuracy and widen the scope of the model for clinical applications. Upon successful implementation, this method could provide a robust and cost-effective tool for noninvasive quantification of skull thickness along with blood flow at the bedside for continuous monitoring of brain function at the clinic.

## Supporting information

S1 Table Fitted *d*_1_, *D*_B2_, and *αD*_*B*_ for all measurements. (DOCX)
