# Supplementary material for: Two-layer analytical model for estimation of layer thickness and flow using Diffuse Correlation Spectroscopy
Source: PLoS One. 2022 Sep 16;17(9):e0274258. doi: 10.1371/journal.pone.0274258 (PMC9481000; doi:10.1371/journal.pone.0274258)
Supplement: S1 Table — (DOCX) [file pone.0274258.s001.docx]

# Two-layer analytical model for estimation of layer thickness and flow using Diffuse Correlation Spectroscopy

Jingyi Wu^1¶^, Syeda Tabassum^1¶^, William L. Brown^1^, Sossena Wood^1^, Jason Yang^1^, Jana M. Kainerstorfer^1,2,*^

^1^ Department of Biomedical Engineering, Carnegie Mellon University, Pittsburgh, Pennsylvania, United States of America

^2^ Neuroscience Institute, Carnegie Mellon University, Pittsburgh, Pennsylvania, United States of America

^*^ Corresponding author

E-mail: jkainers@andrew.cmu.edu (JMK)

^¶^These authors contributed equally to this work.

Short title: Two-layer analytical model for layer thickness and flow using Diffuse Correlation Spectroscopy

**Table 1. Fitted *d*_1_, *D_B_*_2_, and *αD_B_* for all measurements.**

| Source detector separation (SD) | Two-layer phantom | Top layer thickness  d_1_ (mm) | | | Bottom layer Brownian diffusion coefficients (cm^2^⋅s^-1^) | | | |
| --- | --- | --- | --- | --- | --- | --- | --- | --- |
|  |  | True | Fitted | | $D_{B2}$ | | ${\alpha D}_{B}$ | |
|  |  |  | Avg | STD | Avg | STD | Avg | STD |
| SD1  (10 mm) | P0 | 0 | N/A | N/A | 7.89E-09 | 4.23E-11 | 5.85E-09 | 1.04E-10 |
|  | P1 | 2.01 | 3.10 | 0.05 | 5.35E-09 | 9.48E-11 | 2.14E-09 | 5.87E-11 |
|  | P2 | 3.28 | 3.48 | 0.09 | 4.16E-09 | 1.61E-10 | 1.40E-09 | 4.54E-11 |
|  | P3 | 3.87 | 4.03 | 0.13 | 3.31E-09 | 1.74E-10 | 8.36E-10 | 5.60E-11 |
|  | P4 | 4.65 | 4.08 | 0.13 | 2.50E-09 | 3.29E-10 | 6.24E-10 | 5.79E-11 |
|  | P5 | 4.99 | 3.94 | 0.31 | 1.99E-09 | 4.45E-10 | 5.20E-10 | 8.81E-11 |
|  | P6 | 5.96 | 2.59 | 0.67 | 7.67E-10 | 2.75E-10 | 3.46E-10 | 3.60E-11 |
|  | P7 | 8.08 | 1.04 | 0.51 | 2.92E-10 | 5.22E-11 | 2.44E-10 | 1.68E-11 |
| SD2  (15 mm) | P0 | 0 | N/A | N/A | 6.68E-09 | 1.63E-11 | 4.91E-09 | 5.03E-11 |
|  | P1 | 2.01 | 4.71 | 0.16 | 5.72E-09 | 1.55E-10 | 1.81E-09 | 9.40E-11 |
|  | P2 | 3.28 | 5.12 | 0.09 | 4.87E-09 | 2.09E-10 | 1.34E-09 | 2.43E-11 |
|  | P3 | 3.87 | 5.89 | 0.17 | 4.47E-09 | 2.32E-10 | 8.82E-10 | 6.39E-11 |
|  | P4 | 4.65 | 6.23 | 0.13 | 3.99E-09 | 2.29E-10 | 6.64E-10 | 5.02E-11 |
|  | P5 | 4.99 | 5.93 | 0.15 | 3.03E-09 | 5.11E-10 | 5.68E-10 | 7.91E-11 |
|  | P6 | 5.96 | 5.53 | 0.26 | 1.75E-09 | 1.89E-10 | 3.87E-10 | 2.90E-11 |
|  | P7 | 8.08 | 4.45 | 0.36 | 6.86E-10 | 1.26E-10 | 2.29E-10 | 2.12E-11 |
| SD3  (20 mm) | P0 | 0 | N/A | N/A | 6.37E-09 | 2.75E-11 | 4.63E-09 | 5.03E-11 |
|  | P1 | 2.01 | 6.15 | 0.12 | 6.10E-09 | 2.16E-10 | 1.67E-09 | 3.83E-11 |
|  | P2 | 3.28 | 6.89 | 0.11 | 5.83E-09 | 3.12E-10 | 1.22E-09 | 3.54E-11 |
|  | P3 | 3.87 | 7.74 | 0.12 | 5.75E-09 | 3.59E-10 | 8.72E-10 | 3.46E-11 |
|  | P4 | 4.65 | 8.11 | 0.12 | 5.29E-09 | 1.83E-10 | 6.68E-10 | 4.19E-11 |
|  | P5 | 4.99 | 7.74 | 0.25 | 4.36E-09 | 3.24E-10 | 6.47E-10 | 7.85E-11 |
|  | P6 | 5.96 | 7.82 | 0.19 | 3.27E-09 | 3.06E-10 | 4.58E-10 | 4.22E-11 |
|  | P7 | 8.08 | 7.26 | 0.41 | 1.34E-09 | 2.77E-10 | 2.29E-10 | 1.98E-11 |
| SD4  (25 mm) | P0 | 0 | N/A | N/A | 5.99E-09 | 7.00E-11 | 4.30E-09 | 7.14E-11 |
|  | P1 | 2.01 | 7.27 | 0.10 | 5.73E-09 | 1.78E-10 | 1.47E-09 | 3.08E-11 |
|  | P2 | 3.28 | 8.34 | 0.08 | 5.73E-09 | 2.16E-10 | 1.04E-09 | 3.83E-11 |
|  | P3 | 3.87 | 9.18 | 0.11 | 5.68E-09 | 2.71E-10 | 7.70E-10 | 2.23E-11 |
|  | P4 | 4.65 | 9.65 | 0.15 | 5.68E-09 | 4.48E-10 | 6.24E-10 | 4.11E-11 |
|  | P5 | 4.99 | 9.25 | 0.18 | 5.20E-09 | 5.56E-10 | 6.79E-10 | 9.57E-11 |
|  | P6 | 5.96 | 9.46 | 0.19 | 4.01E-09 | 2.36E-10 | 4.69E-10 | 3.09E-11 |
|  | P7 | 8.08 | 9.44 | 0.30 | 2.02E-09 | 3.47E-10 | 2.24E-10 | 2.28E-11 |
